# Catalase Defines Radiotherapy Resistance and a Therapeutic Vulnerability in Rhabdomyosarcoma

**DOI:** 10.3390/ijms27167147

**Published:** 2026-08-10

**Authors:** Silvia Codenotti, Francesco Marampon, Francesca Megiorni, Enrico Romano, Silvia Pomella, Rossella Rota, Isabella Zanella, Eugenia Quiros-Roldan, Giovanni Corsetti, Luca Triggiani, Sara Salucci, Irene Faenza, Martina Benedetti, Mattia Bugatti, William Vermi, Alessandro Fanzani

**Affiliations:** 1 Department of Molecular and Translational Medicine, University of Brescia, Viale Europa 11, 25123 Brescia, Italy; silvia.codenotti@unibs.it (S.C.); isabella.zanella@unibs.it (I.Z.); william.vermi@unibs.it (W.V.); 2 Unit of Pathology, ASST Spedali Civili di Brescia, Piazzale Spedali Civili 1, 25123 Brescia, Italy; marti.benedetti99@icloud.com (M.B.); mattia.bugatti@unibs.it (M.B.); 3 Department of Well-being, Health and Environmental Sustainability, Sapienza University of Rome, Via delle Fontanelle s.n.c., 02100 Rieti, Italy; francesco.marampon@uniroma1.it (F.M.); francesca.megiorni@uniroma1.it (F.M.); 4 Department of Sense Organs, Sapienza University of Rome, Piazzale Aldo Moro 5, 00185 Rome, Italy; enrico.romano@uniroma1.it; 5 Department of Hematology/Oncology, Cell and Gene Therapy, Bambino Gesù Children’s Hospital, IRCCS, Piazza Sant’Onofrio 4, 00165 Rome, Italy; silvia.pomella@uniroma2.it (S.P.); rossella.rota@opbg.net (R.R.); 6 Department of Clinical Sciences and Translational Medicine, University of Rome Tor Vergata, Via Montpellier 1, 00133 Rome, Italy; 7 Medical Genetics Laboratory, Diagnostic Department, ASST Spedali Civili di Brescia, Piazzale Spedali Civili 1, 25123 Brescia, Italy; 8 Division of Infectious and Tropical Diseases, ASST Spedali Civili di Brescia, Piazzale Spedali Civili 1, 25123 Brescia, Italy; maria.quirosroldan@unibs.it; 9 Department of Clinical and Experimental Sciences, University of Brescia, Viale Europa 11, 25123 Brescia, Italy; giovanni.corsetti@unibs.it; 10 Radiation Oncology, ASST Spedali Civili di Brescia, Piazzale Spedali Civili 1, 25123 Brescia, Italy; luca.triggiani@unibs.it; 11 Department of Biomedical and NeuroMotor Sciences (DIBINEM), University of Bologna, Via Luigi Zamboni 33, 40126 Bologna, Italy; sara.salucci@unibo.it (S.S.); irene.faenza2@unibo.it (I.F.)

**Keywords:** rhabdomyosarcoma, catalase, redox homeostasis, radioresistance, Akt signaling

## Abstract

Therapeutic resistance remains a critical obstacle in rhabdomyosarcoma (RMS), the most common pediatric soft tissue sarcoma. Increasing evidence implicates redox adaptation in tumor survival and treatment failure. Here, we investigated the functional role and clinical relevance of catalase, a primary hydrogen peroxide-detoxifying enzyme, in modulating RMS therapeutic response. Integrated transcriptomic analyses revealed that while catalase is overall downregulated in RMS compared to healthy skeletal muscle, elevated expression strongly correlates with high-risk, metastatic disease and poor overall survival. In human RMS cell lines, catalase was detectable, and its pharmacological inhibition using 3-amino-1,2,4-triazole (3-ATA) promoted reactive oxygen species (ROS) accumulation, sensitizing cells to standard chemotherapeutics. Notably, robust catalase upregulation was observed in RMS cell models characterized by intrinsic and acquired radioresistance, with strong immunoreactivity validated on cell-block sections. Immunohistochemical validation across patient specimens revealed generally weak catalase expression in RMS tumors, whereas strong reactivity was observed in a secondary embryonal RMS (ERMS) arisen following chemoradiotherapy for nasopharyngeal carcinoma. Functionally, targeting catalase with 3-ATA restored both radio- and chemosensitivity in radioresistant RMS lines. Furthermore, co-targeting catalase and Akt using sub-toxic doses of 3-ATA and MK-2206 cooperatively enhanced oxidative stress-mediated cytotoxicity in resistant cells. Together, these findings identify catalase as a pivotal driver of adaptive radioresistance and establish dual catalase/Akt targeting as a promising pro-oxidant strategy to overcome radiotherapy resistance in RMS.

## 1. Introduction

Rhabdomyosarcoma (RMS) is the most prevalent pediatric soft tissue sarcoma, accounting for 5–8% of all childhood malignancies [1,2]. Originating from mesenchymal progenitor cells committed to the skeletal muscle lineage, RMS is broadly stratified into fusion-negative (FN-RMS) and fusion-positive (FP-RMS) subtypes based on key driver translocations [3], which correspond to histological embryonal (ERMS) and alveolar (ARMS) subtypes, respectively. FN-RMS is a prototypical RAS-driven malignancy frequently harboring mutations in *NRAS*, *KRAS*, *HRAS*, *NF1*, and *PIK3CA* [4]. Conversely, FP-RMS is characterized by recurrent chromosomal translocations, primarily $t\left(2;13\right)$ or $t\left(1;13\right)$, that yield high-risk PAX3::FOXO1 or PAX7::FOXO1 fusion oncoproteins, respectively [5]. Independent of fusion status, hyperactivation of the PI3K pathway occurs in roughly 80–90% of all RMS tumors, driving cell proliferation and survival [6].

Standard RMS clinical management employs a multimodal approach combining surgery, radiation, and chemotherapy based on vincristine, actinomycin D, and cyclophosphamide (the VAC backbone). Low-risk patients receive vincristine and actinomycin D alone to limit cumulative cyclophosphamide toxicities (such as secondary malignancies and gonadotoxicity), whereas intermediate- and high-risk patients are treated with intensified VAC-based regimens. Although effective against localized primary disease, current therapeutic strategies yield dismal outcomes in metastatic or relapsed settings, where treatment failure is predominantly driven by acquired resistance [7]. 

Accumulating evidence highlights redox homeostasis as a critical determinant of RMS progression and therapeutic resistance [8,9]. Reactive oxygen species (ROS) exhibit context-dependent dual roles in cancer: low-to-moderate ROS levels sustain proliferation and oncogenic signaling, whereas excessive ROS accumulation induces irreversible oxidative damage and triggers cell death [10,11]. To survive chronic oxidative stress, cancer cells upregulate antioxidant defense systems, including catalases, superoxide dismutases (SODs), peroxiredoxins (PRDXs), and glutathione peroxidases (GPXs) [12]. 

In RMS, elevated baseline ROS production is initially sustained by oncogenic RAS signaling, creating a pro-survival feed-forward loop [13]. This high oxidative burden is further compounded by *MCU/MICU1* dysregulation and epigenetic alterations in oxidative stress-related genes (*PTK2*, *COX7A1*, *NOSIP*, *NOS1*, *ATP2A3*, *DDAH1*, *GLRX*, *TXNDC12*) [8], leading to a prominent enrichment of G→T transversions in ERMS. This elevated baseline oxidative stress provides a strong rationale for pro-oxidant therapeutic strategies. Indeed, key backbone drugs like actinomycin D exert their cytotoxicity in part by provoking intracellular ROS surges, inducing lipid peroxidation, and depleting intracellular glutathione (GSH) and SOD reserves. Consequently, pairing pro-oxidant compounds (e.g., HDAC inhibitors) [14], with inhibitors of antioxidant enzymes (e.g., the thioredoxin reductase inhibitor auranofin) [8,15,16] represents a viable therapeutic approach. However, RMS cells frequently develop adaptive mechanisms to scavenge ROS and repair DNA damage. In particular, the PI3K/Akt axis plays a pivotal role in treatment resistance by activating NRF2 to upregulate ABC transporters [17] and stimulating DNA-dependent protein kinase (DNA-PK) [18] to accelerate double-strand break repair [19]. Furthermore, Akt signaling can directly enhance the expression of ROS-scavenging enzymes [20], promoting an oxidative stress-resistant phenotype.

Catalase efficiently converts hydrogen peroxide (H_2_O_2_), a major signaling ROS, into water and oxygen [21,22,23,24]. The functional impact of catalase in oncology is highly context-dependent [25]: while it acts as a tumor suppressor in certain cancers like breast carcinoma [26], it promotes aggressiveness in melanoma, glioblastoma, and Ewing sarcoma [27,28,29]. By buffering cytotoxic ROS surges, high catalase activity frequently confers resistance to both chemo- and radiotherapy [30]. Pharmacological inhibition of catalase using 3-amino-1,2,4-triazole (3-ATA), which covalently inactivates the enzyme’s catalytic site [31], has demonstrated promising therapeutic sensitization in experimental models [32,33].

Despite growing interest in RMS redox biology [8], the specific role of catalase in orchestrating RMS progression and therapy resistance remains uncharacterized. In this study, we investigated catalase expression and function in RMS using transcriptomic datasets, cell-based models, and patient tissue specimens. We demonstrated that while baseline catalase is low in RMS tumors, elevated levels correlate with poor survival and strong upregulation marks post-radiation radioresistance. Pharmacologically targeting catalase, alone via 3-ATA or synergistically alongside Akt signaling, restores chemo- and radiosensitivity through ROS accumulation, establishing catalase as a pivotal target in aggressive RMS. 

## 2. Results

### 2.1. Catalase Is Downregulated in RMS, but Higher Expression Predicts Poor Patient Survival

**Figure 1:**
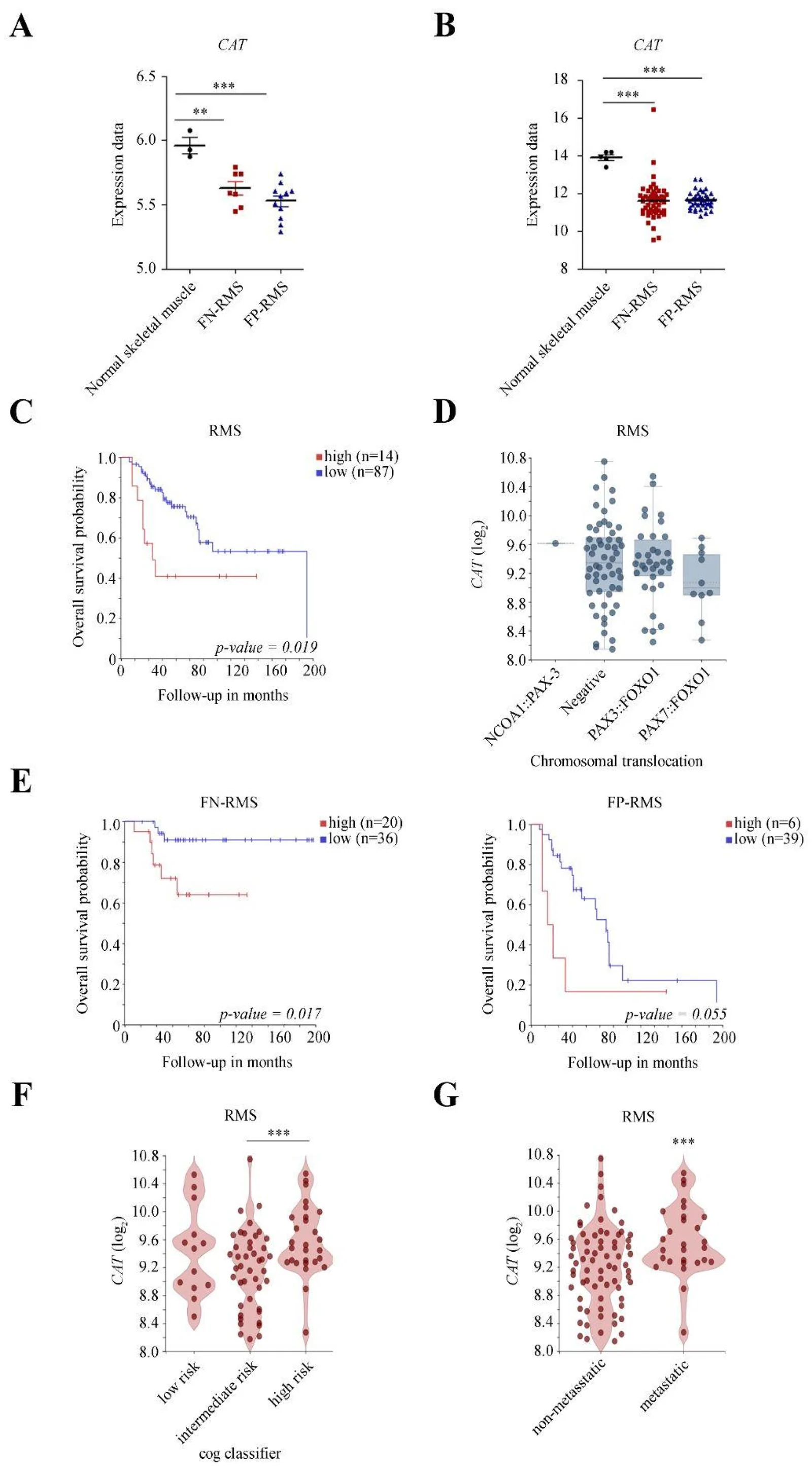
Catalase expression and its prognostic value in RMS: in silico transcriptomic analysis. (**A**,**B**) Quantification of *CAT* gene expression in mouse (**A**) (GSE22520; FN-RMS *n* = 7, FP-RMS *n* = 11, normal skeletal muscle *n* = 5) and human (**B**) (GSE108022; FN-RMS *n* = 66, FP-RMS *n* = 35, normal skeletal muscle *n* = 3) transcriptomic datasets. Individual sample values are plotted, with horizontal lines representing mean values. ** *p*-value < 0.01; *** *p*-value < 0.001 vs. normal skeletal muscle; one-way ANOVA with Dunnett’s post hoc test. (**C**–**G**) *In silico* clinical characterization performed via the R2 platform on a public RMS transcriptomic cohort (*n* = 101; https://www.ebi.ac.uk/biostudies/arrayexpress/studies/E-TABM-1202) (accessed on 19 January 2026). (**C**) Kaplan–Meier overall survival curves of RMS patients stratified by *CAT* transcript levels (high, red; low, blue; log-rank test *p* = 0.019). (**D**) Boxplot displaying *CAT* expression (log_2_) according to driver chromosomal translocations: NCOA1::PAX3 (*n* = 1), fusion-negative (*n* = 56), PAX3::FOXO1 (*n* = 34), and PAX7::FOXO1 (*n* = 10). (**E**) Kaplan–Meier overall survival curves for FN-RMS (left, *n* = 56) and FP-RMS (right, *n* = 45) cohorts stratified by *CAT* expression (log-rank test). (**F**,**G**) Violin plots showing *CAT* expression (log_2_) across Children’s Oncology Group (COG) risk categories (low, *n* = 13; intermediate, *n* = 43; high, *n* = 28) (**F**) and comparing non-metastatic (*n* = 72) vs. metastatic (*n* = 28) cases (**G**). *** *p*-value < 0.001; one-way ANOVA for (**F**) and two-tailed unpaired *t*-test for (**G**).

We first evaluated the baseline expression levels of *CATALASE* (*CAT)* in healthy skeletal muscle *versus* RMS tumor tissue across two independent RNA datasets from mouse (GSE22520) and human (GSE108022) RMS (Figure 1A,B). In the first cohort, *CAT* expression was significantly downregulated in both tumor subtypes compared to normal skeletal muscle, showing a significant reduction in FN-RMS (*p* < 0.01) and a highly significant decrease in FP-RMS (*p* < 0.001; Figure 1A). This drastic reduction (*p* < 0.001) in *CAT* expression across both subtypes was successfully validated in a second, larger cohort (Figure 1B).

We next investigated the clinical relevance of *CAT* expression in RMS. Kaplan–Meier analysis of transcriptomic datasets revealed that high *CAT* expression was significantly associated with reduced overall survival in a cohort of 101 RMS patients (*p* = 0.019; Figure 1C). Although *CAT* expression levels did not differ significantly based on specific chromosomal translocations or between FN and FP subtypes (Figure 1D), high *CAT* levels stratified patients into poorer survival outcomes within both subgroups (FN-RMS, *p* = 0.017; FP-RMS, *p* = 0.055; Figure 1E). Moreover, *CAT* expression was significantly elevated in high-risk compared with intermediate-risk tumors (*p* = 9.61 × 10^−3^; Figure 1F) and in metastatic *versus* non-metastatic cases (*p* = 4.08 × 10^−3^; Figure 1G). Together, these findings indicate that while *CAT* is significantly downregulated in RMS compared to healthy skeletal muscle, its elevated expression within tumor tissues strongly associates with aggressive disease features and unfavorable clinical outcomes.

### 2.2. Catalase Is Expressed in Human FN- and FP-RMS Cell Lines, and Its Inhibition Enhances ROS Accumulation and Chemosensitivity

**Figure 2:**
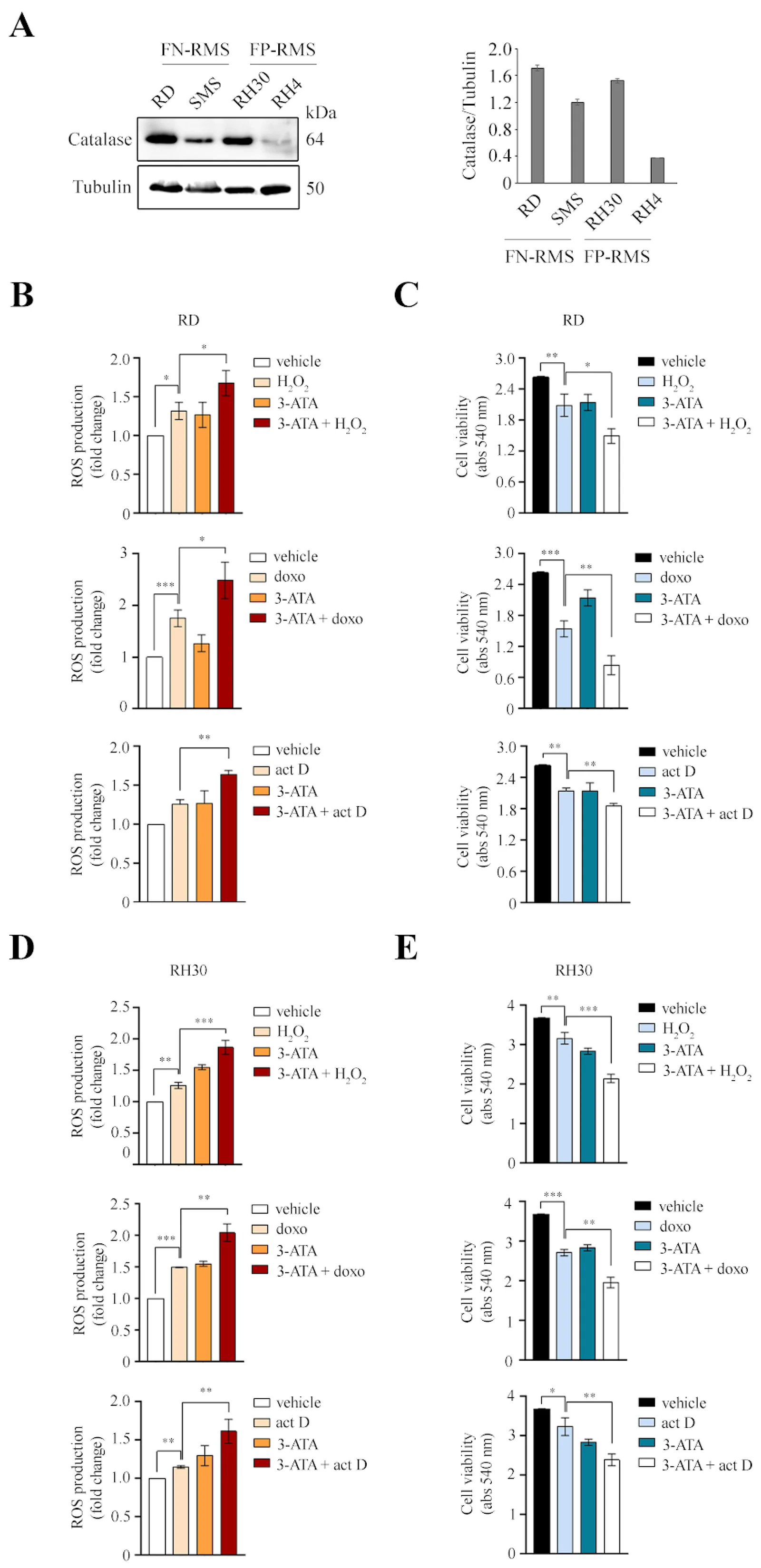
Catalase inhibition increases ROS levels and enhances chemosensitivity in RMS cells. (**A**) Representative immunoblotting analysis of catalase protein expression in FN-RMS (RD, SMS) and FP-RMS (RH30, RH4) cell lines (left). Relative protein levels normalized to tubulin loading control are quantified on the right (*n* = 2). (**B**–**E**) RD (**B**,**C**) and RH30 (**D**,**E**) cells were treated with H_2_O_2_ (200 µM, top panels), doxorubicin (doxo, 1 µM, middle panels), or actinomycin D (act D, 0.5 nM, bottom panels), with or without overnight pretreatment with the catalase inhibitor 3-ATA (50 mM). (**B**,**D**) Intracellular ROS production measured after 24 h using CM-$H_{2}DCFDA\;staining$, expressed as fold change relative to vehicle-treated controls (*n* = 3). (**C**,**E**) Cell viability evaluated after 48 h by MTT assay (absorbance at 540 nm; *n* = 3). Data are presented as mean ± SEM. * *p* < 0.05, ** *p* < 0.01, *** *p* < 0.001; one-way ANOVA followed by Tukey’s post hoc test.

To assess catalase protein levels, we performed immunoblotting analysis on a panel of RMS cell lines, including FN-RMS (RD, SMS) and FP-RMS (RH30, RH4) models (Figure 2A). Catalase protein expression was detected in all examined lines, with RD and RH30 cells exhibiting the highest baseline levels (Figure 2A). To determine the functional role of catalase, RD and RH30 cells were treated with H_2_O_2_, doxorubicin, or actinomycin D, either alone or in combination with the catalase inhibitor 3-ATA. ROS levels and cell viability were assessed after 24 and 48 h, respectively. In RD cells, 3-ATA alone modestly increased ROS levels, whereas co-treatment significantly enhanced the oxidative stress induced by H_2_O_2_, doxorubicin, and actinomycin D (Figure 2B); this synergistic effect on ROS accumulation was particularly evident when combined with actinomycin D. Consistently, 3-ATA significantly potentiated the reduction in cell viability induced by all three pro-oxidant treatments (Figure 2C). A similar pattern was observed in RH30 cells, where catalase inhibition markedly exacerbated ROS accumulation across all conditions, with the most pronounced effect seen in combination with H_2_O_2_ (Figure 2D). This oxidative peak was closely paralleled by a significant decrease in cell viability (Figure 2E). Overall, these data indicate that catalase inhibition efficiently enhances intracellular oxidative stress and sensitizes both FN- and FP-RMS cells to chemotherapeutic agents.

### 2.3. Catalase Upregulation Is Associated with a Radioresistant Cell and Tumor Phenotype

**Figure 3:**
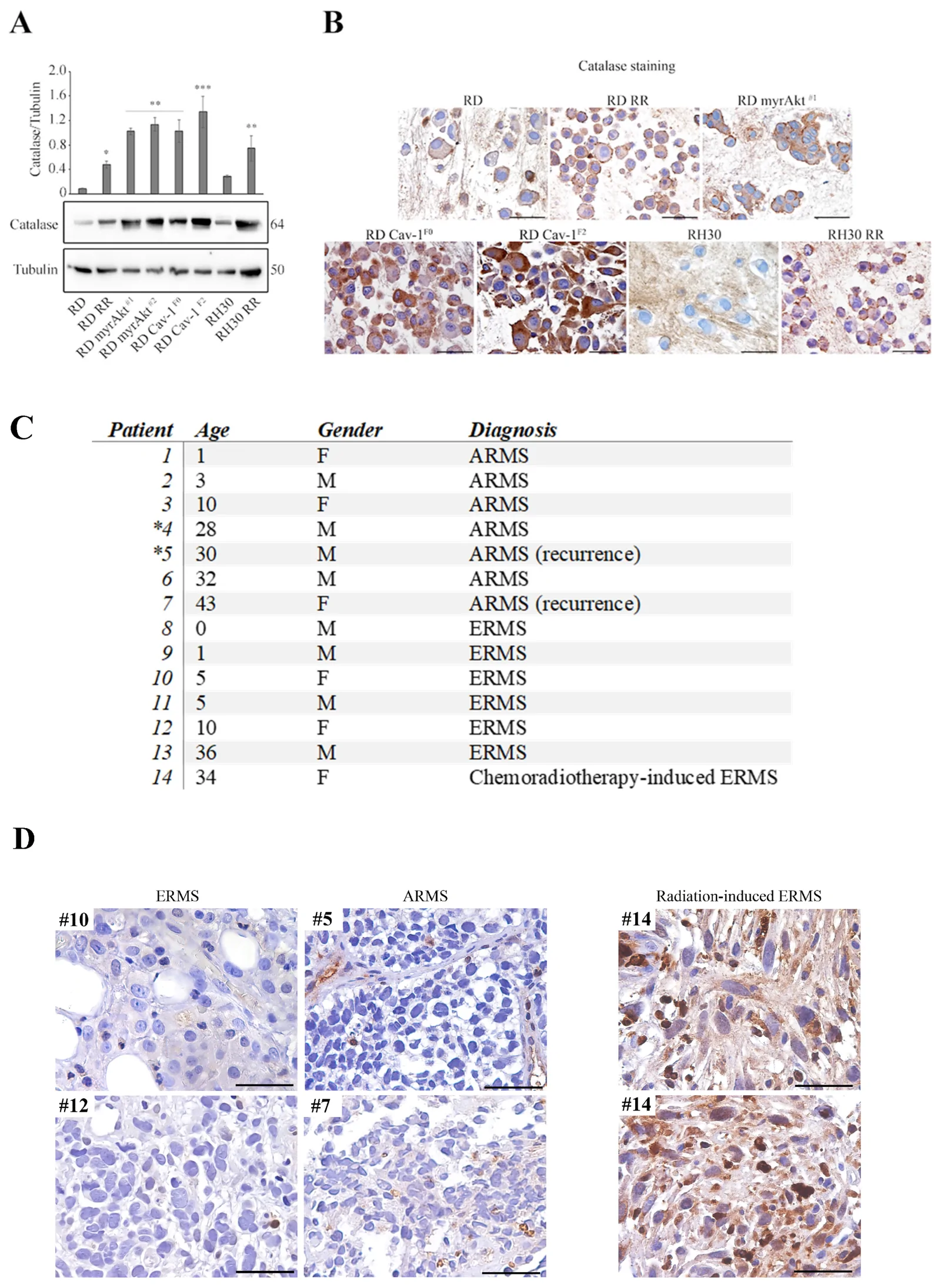
Catalase is upregulated during the acquisition of a radioresistant RMS phenotype. (**A**) Representative immunoblotting analysis of catalase protein expression (**bottom**) and densitometric quantification normalized to tubulin loading control (**top**) in parental (RD, RH30) and radioresistant RMS cell lines (RD RR, RD myrAkt^#1^, and RD myrAkt^#2^, RD Cav-1^F0^, RD Cav-1^F2^, and RH30 RR); n=2. * *p* < 0.05, ** *p* < 0.01, *** *p* < 0.001; one-way ANOVA test. (**B**) Representative immunocytochemical staining of cell-block sections from parental and resistant RMS models displaying cytoplasmic catalase expression (brown) counterstained with hematoxylin (blue) (magnification 400×, scale bar: 50 µm). (**C**) Summary table of patient demographics and clinical diagnoses for the RMS tissue cohort (*F*: Female; *M*: Male; age 0 indicates an infant <1 year old). Asterisks (*) denote sequential/paired samples from the same patient. (**D**) Representative catalase IHC staining in ERMS (samples #10 and #12) and ARMS (samples #5 and #7) specimens (left panel; 200× magnification, scale bar: 50 µm), alongside a post-radiation secondary ERMS specimen (sample #14, two fields) showing strong, diffuse immunoreactivity (right panel; 200× magnification, scale bar: 50 µm). Counterstain: hematoxylin.

In previous studies, we generated diverse radioresistant RMS models utilizing distinct experimental strategies. Specifically, we established models of intrinsic radioresistance in FN-RMS RD cells via the overexpression of either a constitutively active Akt1 (RD myrAkt) [18] or Caveolin-1 (RD Cav-1) [34,35], both of which activate pathways that promote ROS detoxification and DNA repair. In parallel, we utilized FN- and FP-RMS models of acquired radioresistance (RD RR and RH30 RR), which were generated through chronic, fractionated irradiation up to a cumulative dose of 66 Gy [36]. Immunoblotting analysis revealed that all these independent radioresistant cell models exhibited significantly higher endogenous catalase expression compared to their respective parental lines (Figure 3A). Accordingly, the expression pattern of catalase was confirmed in cell-block sections across all radioresistant models, with RD RR, RD myrAkt, RD Cav-1 and RH30 RR displaying markedly elevated catalase staining compared to parental RD and RH30 cells (Figure 3B). Consistent with the immunoblotting data, the highly metastatic RD Cav-1^F2^ clone demonstrated the highest catalase expression levels (Figure 3A,B). To extend our findings to a clinical context, we evaluated catalase expression in a cohort of RMS specimens, encompassing both the ARMS (samples #1-7) and ERMS (samples #8-13) subtypes (Figure 3C). Additionally, we examined an illustrative clinical case of secondary ERMS (sample #14) that arose in a patient following chemo- and radiotherapy for nasopharyngeal carcinoma (detailed in the Materials and Methods). In tumors, immunohistochemistry (IHC) analysis revealed that catalase expression was weak or undetectable across most cases, regardless of histological subtype or recurrent status (Figure 3D, left panel). Notably, the minor catalase expression observed within these primary tissues was predominantly localized to non-tumoral populations in the microenvironment, particularly infiltrating macrophages (Figure 3D, left panel). This relative absence of catalase in primary RMS cells aligns with our *in silico* observations, which demonstrated reduced *CAT* expression in RMS patient samples and mouse models compared to normal skeletal muscle (Figure 1A,B). Strikingly, whereas RMS specimens exhibited negligible tumor catalase staining, the ERMS specimen arising post-chemoradiotherapy displayed strong, diffuse cytoplasmic immunoreactivity for catalase throughout the malignant cells (Figure 3D, right panel). Although based on an initial proof-of-concept clinical observation, these findings closely mirror our in vitro data, suggesting that catalase induction may serve as a pivotal adaptive mechanism promoting tumor survival under radiation-induced oxidative stress.

### 2.4. Catalase Inhibition Restores Radio- and Chemosensitivity in Resistant RMS Models, and Dual Inhibition with Akt Potentiates the Therapeutic Response

**Figure 4:**
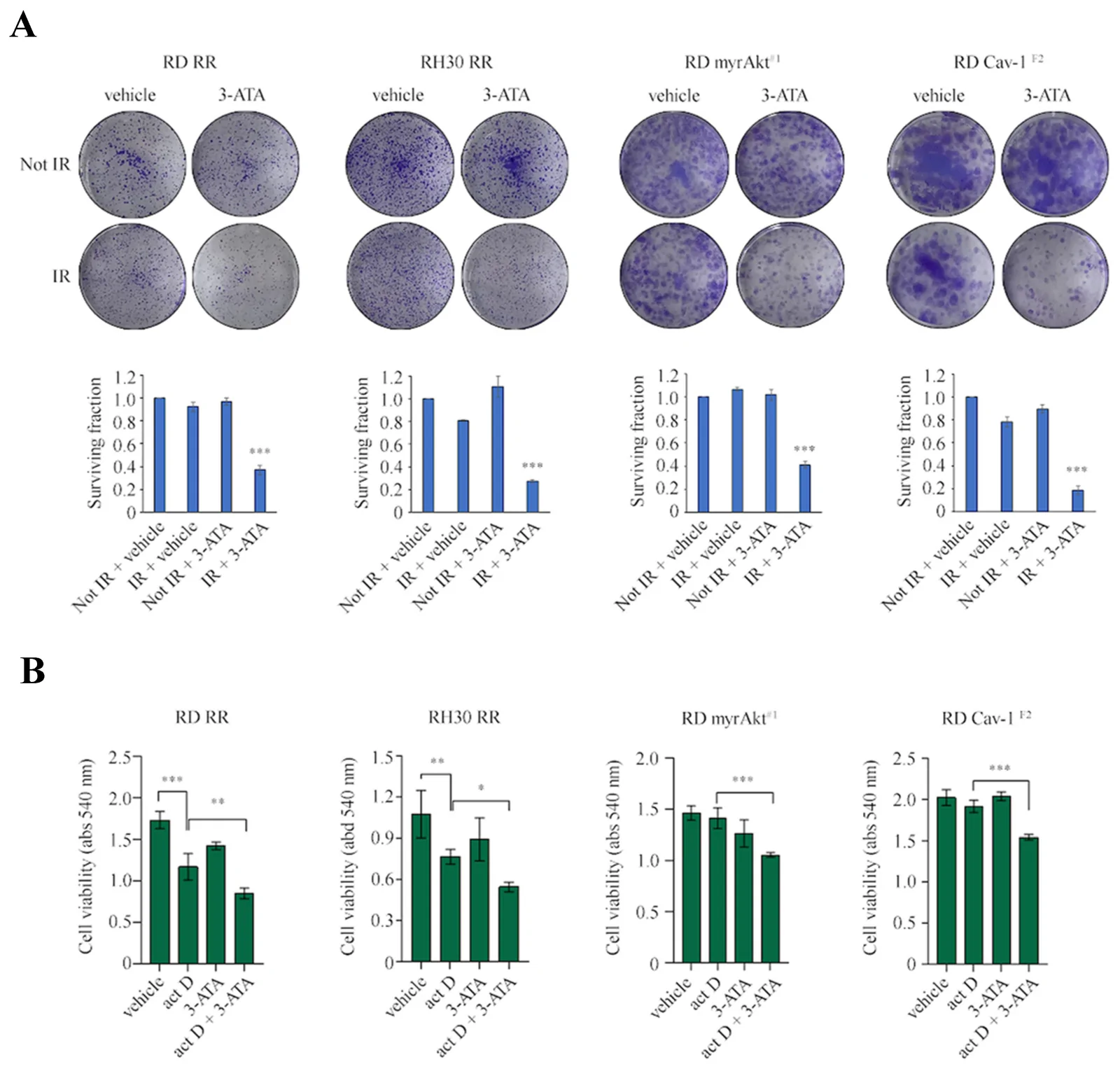
Catalase inhibition restores radio- and chemosensitivity in resistant RMS models. (**A**) Clonogenic survival assay in the indicated radioresistant RMS cell lines (RD RR, RH30 RR, RD myrAkt#1, and RD Cav-1^F2^) pretreated overnight with 3-ATA (50 mM) or vehicle control prior to IR (4 Gy). Representative colony wells stained with crystal violet after 10–14 days (**top**) and quantification of the surviving fraction relative to control (**bottom**; n=3). (**B**) Cell viability evaluated by MTT assay (absorbance at 540 nm) after 48 h of treatment with actinomycin D (act D, 0.5 nM), with or without overnight pretreatment with 3-ATA (50 mM; *n* = 3). Data are presented as mean ± SEM. * *p* < 0.05, ** *p* < 0.01, *** *p* < 0.001; one-way ANOVA followed by Tukey’s post hoc test.

**Figure 5:**
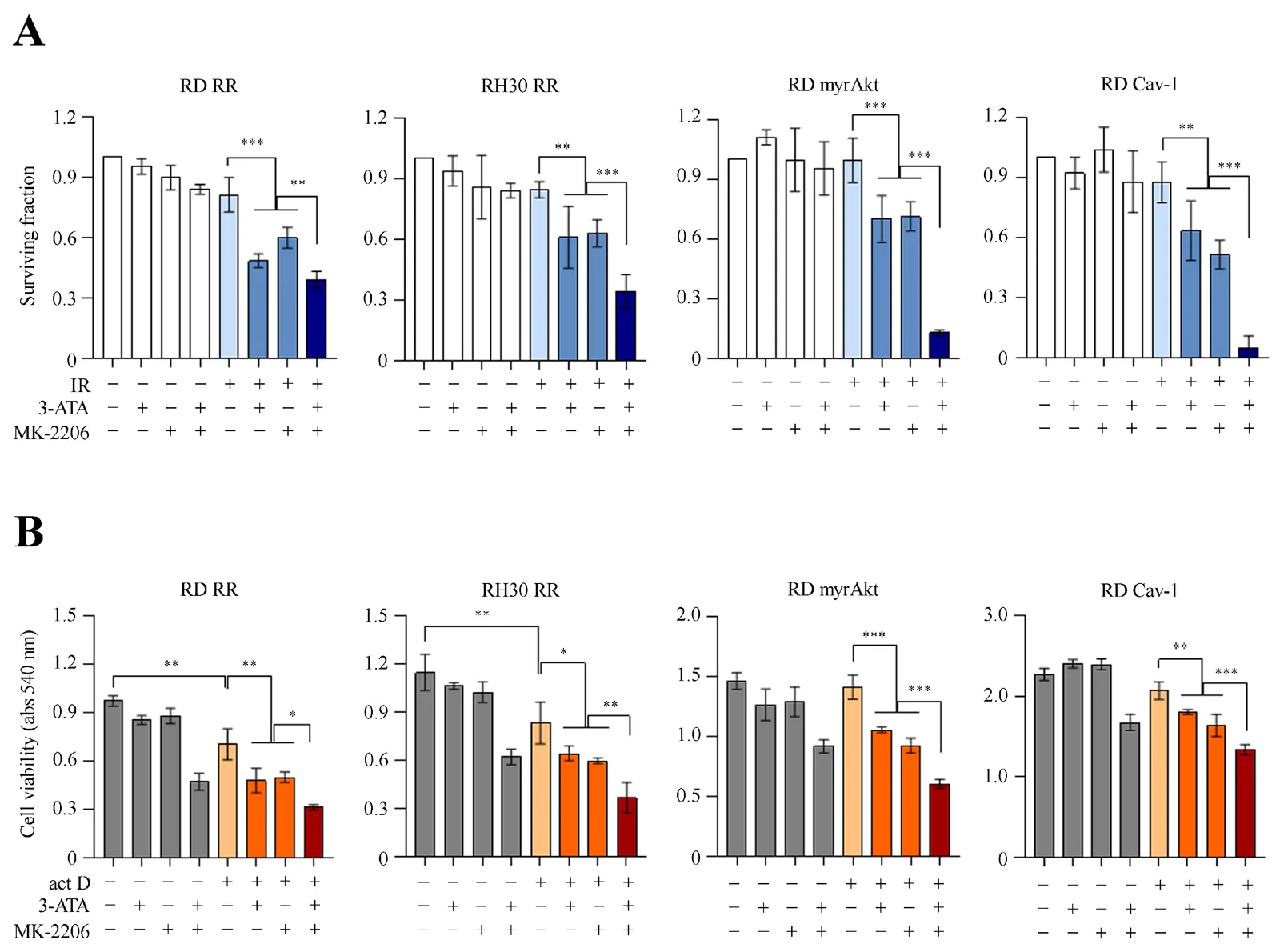
Combined inhibition of catalase and Akt enhances therapeutic response in radioresistant RMS models. (**A**) Clonogenic survival assay in radioresistant RMS cell lines (RD RR, RH30 RR, RD myrAkt, and RD Cav-1) pretreated with 3-ATA (50 mM), the Akt inhibitor MK-2206 (1 µM), or their combination, in the presence (colored bars) or absence (white bars) of IR (4 Gy). Histograms display the quantification of surviving fractions relative to untreated controls (*n* = 3). (**B**) Cell viability evaluated by MTT assay (absorbance at 540 nm) after 48 h of treatment with actinomycin D (act D, 0.5 nM), 3-ATA (50 mM), MK-2206 (1 µM), or their combinations in the presence of chemotherapy (colored bars), alongside baseline combination treatments in the absence of chemotherapy (grey bars) (*n* = 3). Data are presented as mean ± SEM. * *p* < 0.05, ** *p* < 0.01, *** *p* < 0.001; one-way ANOVA followed by Tukey’s post hoc test.

We sought to determine whether targeting catalase could reverse radioresistance. While radioresistant sublines displayed limited sensitivity to ionizing radiation (IR) alone, pharmacological pretreatment with the catalase inhibitor 3-ATA significantly impaired clonogenic survival across all tested models, as evidenced by a drastic reduction in both colony-forming ability and surviving fractions (Figure 4A). In parallel, we evaluated whether catalase inhibition could similarly overcome chemoresistance. Although actinomycin D mono-treatment exerted limited cytotoxicity in several resistant lines, its combination with 3-ATA consistently and significantly synergized to reduce cell viability across all four radioresistant models (Figure 4B). The Akt pathway is overactivated in approximately 93% of RMS cases [4], playing a pivotal role in tumor survival and being consistently upregulated in our chemo- and radioresistant models. Although Akt inhibitors exhibit efficacy against RMS, their clinical translation is frequently hampered by dose-limiting toxicities. To address this limitation, we investigated whether dual targeting of catalase and Akt signaling could offer a more effective and viable therapeutic strategy. While IR alone had a minimal impact on the clonogenic survival of these resistant lines, single-agent treatment with either 3-ATA or the Akt inhibitor MK-2206 led to a modest reduction in colony formation (Figure 5A). Notably, the simultaneous combination of both inhibitors at sub-toxic doses resulted in a significantly greater reduction in clonogenic survival upon irradiation across all radioresistant models, with the most profound sensitization observed in RD myrAkt and RD Cav-1 cells (Figure 5A). Similarly, dual inhibition successfully enhanced tumor cell sensitivity to actinomycin D. While single-agent treatments moderately decreased cell viability, combining 3-ATA and MK-2206 exerted a strikingly more pronounced cytotoxic effect when paired with actinomycin D in all tested models (Figure 5B). Interestingly, baseline combined treatment with 3-ATA and MK-2206 (in the absence of radiation or chemotherapy) significantly reduced cell viability in short-term MTT assays in radioresistant models such as RD RR and RH30 RR (Figure 5B, grey bars). In contrast, its impact on long-term colony-forming ability was less pronounced under non-irradiated conditions (Figure 5A, white bars), where robust synergistic lethality was unmasked only upon combination with IR or chemotherapy (Figure 5A,B, blue and orange/red bars). This divergence likely reflects differences in treatment duration, assay endpoints (metabolic activity vs. reproductive integrity), or the capacity of single cells to recover over extended periods. Collectively, our results demonstrate that catalase inhibition restores both radio- and chemosensitivity in treatment-resistant RMS cells. Furthermore, dual inhibition of catalase and Akt cooperatively enhances oxidative stress-mediated cytotoxicity, effectively overcoming therapy resistance in aggressive RMS models.

## 3. Discussion

Catalase regulates H_2_O_2_ availability and exerts context-dependent effects on tumor growth and metastatic potential [30]. Here, we identify catalase as a central node linking redox adaptation, therapy resistance, and clinical aggressiveness in RMS. We show for the first time that elevated *CAT* expression correlates with poor clinical outcome, including high-risk disease, metastasis, and reduced overall survival. This clinical association is strongly supported by our experimental models: catalase expression was consistently elevated across multiple independent RMS cell lines characterized by aggressive features, such as Akt hyperactivation [18], Cav-1 overexpression [34,35], and acquired radioresistance following chronic fractionated irradiation [36]. Notably, the highest catalase levels were observed in RD Cav-1^F2^ clones, which are highly metastatic [34], suggesting that catalase upregulation represents a hallmark adaptive feature of RMS progression. 

A remarkable finding of our study is that catalase upregulation occurs universally across both intrinsic (RD myrAkt and RD Cav-1) and acquired (RD RR and RH30 RR) radioresistant models, encompassing both FN- and FP-RMS subtypes. This reflects a conserved, subtype-independent mechanism of redox homeostasis. This convergence underscores a shared survival strategy wherein RMS cells, regardless of their driving genetic background or mode of resistance, rely on the induction of key antioxidant enzymes like catalase to buffer oxidative stress and evade apoptosis. Notably, acquired radioresistant sublines (RD RR and RH30 RR) were previously shown to harbor elevated endogenous levels and activation of both Akt and Cav-1 [35], pointing to an adaptive survival network engaged by chronic radiation pressure. Consequently, our genetically engineered intrinsic models, driven by the singular overexpression of Akt1 or Cav-1, faithfully recapitulate key elements of this acquired radioresistance program, positioning catalase as a central convergence node of therapy resistance. This suggests that the Akt/Cav-1/catalase signaling axis is a valuable therapeutic target in RMS.

The molecular mechanisms regulating catalase expression in cancer remain incompletely defined. Several transcription factors, including Sp1, NF-Y, and CCAAT/enhancer-binding protein-β (C/EBP-β), are known to engage regulatory elements within the catalase promoter [37,38,39]. Notably, NF-Y-dependent transcription can be augmented by endoplasmic reticulum (ER) stress through activation of the unfolded protein response (UPR) [40,41]. Because IR triggers both chronic ER stress and sustained oxidative burden [42], and RMS cells exhibit high baseline UPR signaling [43,44,45,46], this pathway represents a plausible mechanism driving catalase upregulation in response to radiation or intrinsic stress. Our radioresistant cell models are all characterized by elevated Akt signaling, suggesting that catalase may be under control of this pathway, as observed in MCF-7 breast cancer cells [47]. Whether catalase represents a direct target of Akt signaling in RMS warrants further investigation. Beyond transcription factor control, catalase expression is subject to epigenetic regulation [48,49]. Under chronic oxidative stress, we hypothesize that both transcriptional and epigenetic mechanisms drive the upregulation of catalase in RMS.

Crucially, our study highlights the dual behavior of catalase in tumor progression: while globally downregulated in primary tumors compared to healthy skeletal muscle, it is markedly re-expressed under therapeutic stress. Indeed, IHC analysis of 11 primary and two recurrent RMS samples revealed weak or undetectable baseline catalase staining. In striking contrast, robust catalase positivity was observed in a clinical specimen of secondary ERMS that developed in the nasal cavity of a 34-year-old woman, 15 years after chemoradiotherapy for nasopharyngeal carcinoma. We hypothesize that prior therapeutic pressure selected for a resilient cell population, culminating in a secondary ERMS equipped with high catalase-mediated antioxidant protection. This histological evidence, although preliminary, provides a pivotal bridge between our *in silico* predictions and *in vitro* findings. Although our mechanistic characterization relies primarily on cell-based genetic and pharmacological models, this clinical IHC case provides a critical first-in-human proof of concept. Formal validation in expanded, prospective clinical cohorts will be essential to confirm these preliminary results.

Pharmacological inhibition of catalase using 3-ATA [50,51] successfully restored both chemo- and radiosensitivity in radioresistant RMS models through ROS-dependent mechanisms, mirroring findings in other tumor types [52]. To maximize the translational impact of this approach, we evaluated a novel combinatorial regimen. Dual targeting of catalase and Akt, a key regulator of cell survival and redox homeostasis [17,53,54,55], yielded synergistic oxidative stress-induced apoptosis across all RMS models. Remarkably, this synergistic cytotoxic effect was achieved using sub-toxic doses approximately five-fold (3-ATA) and ten-fold (MK-2206) lower than their respective single-agent IC50 values, highlighting a targetable dependency between antioxidant defense and Akt signaling that can be exploited to widen the therapeutic window. Moving forward, evaluating the safety, pharmacokinetics, and in vivo efficacy of this combination will be essential for clinical translation.

Given the intrinsically high baseline oxidative stress of RMS cells [8,56,57], combining catalase inhibition with standard-of-care regimens (such as VAC chemotherapy) or pro-oxidant therapies (e.g., auranofin, erastin, or buthionine sulfoximine) represents a highly rational therapeutic strategy [8,14,15,16,58,59,60,61,62,63,64]. Nevertheless, key limitations of our study should be noted. Although 3-ATA effectively targets catalase *in vitro*, its translational utility is constrained by off-target reactivity and potential rodent carcinogenicity [32,33], highlighting the need for selective, next-generation catalase inhibitors. The current landscape of small-molecule catalase inhibitors remains narrow, consisting predominantly of agents that target key active-site residues or NADPH cofactor binding. Examples include the *Pseudomonas aeruginosa* toxin pyocyanin [65], the benzaldehyde thiosemicarbazone derivative BT-Br66, and various natural flavonoids (such as myricetin, epicatechin gallate, and quercetin) [66] or phenolic acids [67]. Alternative strategies, including 400–420 nm blue light photoinactivation, used to deplete catalase in resistant bacteria [68], have not yet been translated to oncology. Given these constraints, future studies should evaluate novel highly specific small molecules or utilize genetic loss-of-function models (e.g., siRNA or CRISPR-Cas9 knockout) to definitively isolate the biological consequences of catalase depletion in RMS.

In summary, our findings establish catalase as both a clinical biomarker of treatment-adapted disease and a therapeutically actionable vulnerability in RMS. Disrupting catalase-driven redox adaptation, particularly when combined with Akt pathway inhibition, represents a potent strategy to overcome therapy resistance and improve clinical outcomes in aggressive RMS.

## 4. Materials and Methods

### 4.1. In Silico Analysis

Bioinformatic analyses were performed using a mouse dataset (GSE22520) of 7 FN-RMS, 11 FP-RMS, and 5 skeletal muscle samples; a human dataset (GSE108022) of 66 FN-RMS, 35 FP-RMS, and 3 skeletal muscle samples; and a cohort of 101 RMS patients from the publicly available dataset E-TABM-1202 (https://www.ebi.ac.uk/biostudies/arrayexpress/studies/E-TABM-1202 (accessed on 19 January 2026)), in which tumors were classified as FN-RMS (n = 56) and FP-RMS (n = 45). The latter included PAX3::NCOA1 (n = 1), PAX3::FOXO1 (n = 34), and PAX7::FOXO1 (n = 10) transcript fusion samples. Kaplan–Meier survival analyses and correlation analyses between gene expression and clinical parameters were generated using the R2 platform (https://hgserver1.amc.nl/ (accessed on 19 January 2026)).

### 4.2. Cell Cultures

Human RD and RH30 RMS cell lines were obtained from the European Collection of Cell Cultures (ECACC, Salisbury, UK). Human SMS-CTR and RH4 were kindly provided by Prof. Maurizio Onisto (University of Padova, Italy). Stable myrAkt1- and Cav-1-overexpressing clones were generated as previously described [18,34]. Radioresistant RD and RH30 cell lines were obtained as described in [36]. Cells were maintained in high-glucose Dulbecco’s Modified Eagle’s Medium (DMEM; Euroclone, Milan, Italy) supplemented with 10% fetal bovine serum (FBS; Euroclone), 100 μg/mL penicillin/streptomycin (Merck, Milan, Italy), and 1 mM L-glutamine (Merck), in a humidified incubator at 37 °C with 5% CO_2_.

### 4.3. Antibodies

Primary antibodies against catalase and α-tubulin were obtained from Santa Cruz Biotechnology (Dallas, TX, USA) and Merck, respectively. HRP-conjugated anti-mouse and anti-rabbit secondary antibodies were purchased from Immunological Sciences (Rome, Italy).

### 4.4. Immunoblotting

Cells were lysed in cold RIPA buffer (20 mM Tris-HCl at pH 7.6, 1% Nonidet P40, 0.5% sodium deoxycholate, 0.1% SDS, 50 mM NaCl) supplemented with phosphatase inhibitors (1 mM Na_3_VO_4_ and 4 mM NaF) and a cocktail of protease inhibitors (Roche, Milan, Italy), sonicated, and centrifuged (12,000× *g*, 10 min, 4 °C). Protein concentration was determined using the Bradford assay. Equal amounts of protein (40 μg) were boiled at 95 °C for 5 min before separation by SDS-PAGE and subsequent transfer onto polyvinylidene fluoride (PVDF) membranes. Membranes were blocked in Tris-buffered saline (TBS) with 0.1% Tween-20 (TBS-T) containing 5% non-fat milk (15 min at room temperature) and incubated with primary antibody (overnight at 4 °C), followed by HRP-conjugated secondary antibody (1 h at room temperature). Signals were detected using an enhanced chemiluminescence reagent (GeneSpin, Milan, Italy). Band intensities were quantified using the Gel Pro Analyzer 4 software (MediaCybernetics Inc., Rockville, MD, USA) and expressed in arbitrary density units.

### 4.5. Cell-Block Preparation

For cell-block preparation, at least 1 × 10^6^ cells were harvested and processed as follows. Cell suspensions of RMS cell lines were centrifuged for 5 min at 800× *g*. A mixture of human plasma (100 µL, kindly provided by the Centro Trasfusionale, ASST Spedali Civili di Brescia) and HemosIL RecombiPlasTin 2G (200 µL, Instrumentation Laboratory, Bedford, MA, USA; cat. no. 0020003050; 1:2 ratio) was added to the cell pellets and mixed until a clot formed, which was then placed into a labeled cassette (Bio-Optica, Milan, Italy; cat. no. 07-7350). Samples were fixed in 10% neutral buffered formalin (Bio-Optica; cat. no. 05-K01004) for 1 h, followed by paraffin embedding. Four-micrometer-thick cell-block sections were processed for IHC.

### 4.6. Cell Treatments

The compounds 3-ATA, H_2_O_2_, and actinomycin D were purchased from Merck. Doxorubicin was obtained from Thermo Fisher Scientific (Milan, Italy), and MK-2206 was from Aurogene (Rome, Italy). 3-ATA was dissolved in deionized water, while doxorubicin and actinomycin D were dissolved in dimethyl sulfoxide (DMSO) as vehicle. Compounds were used at the concentrations indicated in the figure legends. IR treatment was performed using an X-ray linear accelerator at room temperature on subconfluent cells pretreated (or not) with the indicated compounds for the indicated times at 4 Gy (dose rate of 2 Gy/min). Post-irradiation cells were processed for clonogenic assay.

### 4.7. ROS Measurement

The production of reactive oxygen species (ROS) was quantified using the fluorescent probe CM-H_2_DCFDA (Thermo Fisher Scientific), according to the manufacturer’s instructions. Cells were plated in black 96-well plates (2.5 × 10^3^ cells/well) and pretreated with 3-ATA or vehicle overnight. Twenty-four hours after seeding, cells were treated with 200 µM H_2_O_2_, 1 µM doxorubicin, 0.5 nM actinomycin D, or vehicle. After 24 h, the supernatant was removed, and cells were incubated with culture medium containing 1 µM probe for 30 min at 37 °C, protected from light. The probe-containing medium was then replaced with fresh medium. Fluorescence was measured using the EnSight Multimode plate reader (PerkinElmer, Waltham, MA, USA) at 492 nm excitation and 517 nm emission wavelengths. Cells were fixed in paraformaldehyde (PFA) in PBS for 20 min at 4 °C. After fixation, cells were washed with deionized water to remove residual fixative. Bound dye was solubilized using a 1% sodium dodecyl sulfate (SDS) solution in PBS, and plates were shaken continuously until complete dissolution was achieved. Absorbance was subsequently measured at 595 nm using a microplate reader. Results were expressed as the ratio of ROS fluorescence to crystal violet absorbance and reported as fold change relative to the control (set to 1).

### 4.8. MTT Cell Viability Assay

Cells were seeded in 96-well plates (1.5 × 10^3^ cells/well) and pretreated overnight with 3-ATA or vehicle. Twenty-four hours post-seeding, cells were treated with 200 µM H_2_O_2_, 1 µM doxorubicin, 0.5 nM actinomycin D, or vehicle control. Following a 48 h treatment period, the medium was replaced with fresh medium containing 0.5 mg/mL 3-(4,5-dimethylthiazol-2-yl)-2,5-diphenyltetrazolium bromide (MTT), and cells were incubated for 3.5 h at 37 °C. The resulting formazan crystals were solubilized in DMSO, and absorbance was measured at 540 nm using a microplate reader.

### 4.9. Clonogenic Assay

Four hours after irradiation, cells were re-seeded in triplicates into 6-well plates (1000 cells/well) to evaluate clonogenic survival. Colonies were allowed to grow for 10 days, then fixed with a 3% PFA solution (20 min, 4 °C) and stained with crystal violet solution (10 min at room temperature). Images of colonies were acquired after washing with deionized water. The surviving fraction was calculated by dividing the number of colonies by the number of seeded cells, normalized to the plating efficiency of non-irradiated control cells (set to 1).

### 4.10. Immunohistochemistry

Formalin-fixed, paraffin-embedded (FFPE) tissue blocks used in this study were retrieved from the tissue bank of the Department of Pathology, ASST Spedali Civili di Brescia (Brescia, Italy). Four-micrometer-thick tissue sections were prepared for IHC analysis. The analyzed series included 7 ARMS (samples #1-7), 6 ERMS (samples #8-13), and one case of chemoradiotherapy-induced ERMS (sample #14) arising in the nasal cavity of a 34-year-old woman 15 years after standard chemoradiotherapy for nasopharyngeal carcinoma. Catalase expression was evaluated by IHC using a mouse monoclonal anti-catalase antibody (clone H-9, Santa Cruz Biotechnology) at a dilution of 1:70. Antigen retrieval was performed using citrate buffer (pH 6.0). Immunoreactivity was detected using the Novolink Polymer detection system (Leica Microsystems, Milan, Italy), followed by visualization with 3,3′-diaminobenzidine (DAB). Sections were counterstained with hematoxylin.

### 4.11. Statistical Analysis

Data are presented as mean ± SEM. Statistical significance was assessed using an unpaired Student’s *t*-test for pairwise comparisons, or one-way ANOVA test with Tukey’s post hoc test to compare means among three or more groups using GraphPad Prism 8 software (GraphPad Software, San Diego, CA, USA). A *p*-value < 0.05 was considered statistically significant.

## Data Availability

*In silico* analysis was performed by using the publicly available dataset E-TABM-1202 (https://www.ebi.ac.uk/biostudies/arrayexpress/studies/E-TABM-1202 (accessed on 19 January 2026)).

## References

[1] Skapek S.X., Ferrari A., Gupta A.A., Lupo P.J., Butler E., Shipley J., Barr F.G., Hawkins D.S. (2019). Rhabdomyosarcoma. Nat. Rev. Dis. Prim..

[2] Martin-Giacalone B.A., Weinstein P.A., Plon S.E., Lupo P.J. (2021). Pediatric Rhabdomyosarcoma: Epidemiology and Genetic Susceptibility. J. Clin. Med..

[3] Shern J.F., Selfe J., Izquierdo E., Patidar R., Chou H.C., Song Y.K., Yohe M.E., Sindiri S., Wei J., Wen X. (2021). Genomic Classification and Clinical Outcome in Rhabdomyosarcoma: A Report From an International Consortium. J. Clin. Oncol..

[4] Shern J.F., Chen L., Chmielecki J., Wei J.S., Patidar R., Rosenberg M., Ambrogio L., Auclair D., Wang J., Song Y.K. (2014). Comprehensive genomic analysis of rhabdomyosarcoma reveals a landscape of alterations affecting a common genetic axis in fusion-positive and fusion-negative tumors. Cancer Discov..

[5] Marshall A.D., Grosveld G.C. (2012). Alveolar rhabdomyosarcoma-The molecular drivers of PAX3/7-FOXO1-induced tumorigenesis. Skelet. Muscle.

[6] Stevens B.T., Hatley M.E. (2025). Developmental Heterogeneity of Rhabdomyosarcoma. Cold Spring Harb. Perspect. Med..

[7] Wasti A.T., Bisogno G., Hladun R., Defachelles A.S., Casanova M., Breunis W.B., Gatz S.A., Schoot R.A., Ferrari A., Jenney M. (2025). Childhood, Adolescent and Young Adult Poor-Prognosis Rhabdomyosarcoma. Cancers.

[8] Chen X., Stewart E., Shelat A.A., Qu C., Bahrami A., Hatley M., Wu G., Bradley C., McEvoy J., Pappo A. (2013). Targeting oxidative stress in embryonal rhabdomyosarcoma. Cancer Cell.

[9] Pal A., Chiu H.Y., Taneja R. (2019). Genetics, epigenetics and redox homeostasis in rhabdomyosarcoma: Emerging targets and therapeutics. Redox Biol..

[10] Perillo B., Di Donato M., Pezone A., Di Zazzo E., Giovannelli P., Galasso G., Castoria G., Migliaccio A. (2020). ROS in cancer therapy: The bright side of the moon. Exp. Mol. Med..

[11] Reczek C.R., Chandel N.S. (2017). The Two Faces of Reactive Oxygen Species in Cancer. Annu. Rev. Cancer Biol..

[12] Panieri E., Santoro M.M. (2016). ROS homeostasis and metabolism: A dangerous liason in cancer cells. Cell Death Dis..

[13] Zhang M., Linardic C.M., Kirsch D.G. (2013). RAS and ROS in rhabdomyosarcoma. Cancer Cell.

[14] Hedrick E., Crose L., Linardic C.M., Safe S. (2015). Histone Deacetylase Inhibitors Inhibit Rhabdomyosarcoma by Reactive Oxygen Species-Dependent Targeting of Specificity Protein Transcription Factors. Mol. Cancer Ther..

[15] Habermann K.J., Grünewald L., van Wijk S., Fulda S. (2017). Targeting redox homeostasis in rhabdomyosarcoma cells: GSH-depleting agents enhance auranofin-induced cell death. Cell Death Dis..

[16] Kinoshita H., Kinoshita S., Kamoda H., Hagiwara Y., Ohtori S., Yonemoto T. (2024). Thioredoxin Reductase Inhibitor Suppresses the Local Progression of Rhabdomyosarcoma with PDX Models. Cancer Genom. Proteom..

[17] Yang Q., Wang Y., Corchete Sanchez L.A., Mathavarajah S., Qin Q., Wei Y., Alpert E., Whelton L., Sharma P., Strom S. (2025). The PIK3CA/AKT pathway drives therapy resistance in rhabdomyosarcoma. Nat. Commun..

[18] Codenotti S., Zizioli D., Mignani L., Rezzola S., Tabellini G., Parolini S., Giacomini A., Asperti M., Poli M., Mandracchia D. (2022). Hyperactive Akt1 Signaling Increases Tumor Progression and DNA Repair in Embryonal Rhabdomyosarcoma RD Line and Confers Susceptibility to Glycolysis and Mevalonate Pathway Inhibitors. Cells.

[19] Toulany M., Lee K.J., Fattah K.R., Lin Y.F., Fehrenbacher B., Schaller M., Chen B.P., Chen D.J., Rodemann H.P. (2012). Akt promotes post-irradiation survival of human tumor cells through initiation, progression, and termination of DNA-PKcs-dependent DNA double-strand break repair. Mol. Cancer Res..

[20] Codenotti S., Sandrini L., Mandracchia D., Lorenzi L., Corsetti G., Poli M., Asperti M., Salvi V., Bosisio D., Monti E. (2024). Statin-Sensitive Akt1/Src/Caveolin-1 Signaling Enhances Oxidative Stress Resistance in Rhabdomyosarcoma. Cancers.

[21] Nagem R.A., Martins E.A., Gonçalves V.M., Aparício R., Polikarpov I. (1999). Crystallization and preliminary X-ray diffraction studies of human catalase. Acta Crystallogr. D Biol. Crystallogr..

[22] Rhee S.G. (2006). Cell signaling. H2O2, a necessary evil for cell signaling. Science.

[23] Heck D.E., Shakarjian M., Kim H.D., Laskin J.D., Vetrano A.M. (2010). Mechanisms of oxidant generation by catalase. Ann. N. Y. Acad. Sci..

[24] Glorieux C., Calderon P.B. (2017). Catalase, a remarkable enzyme: Targeting the oldest antioxidant enzyme to find a new cancer treatment approach. Biol. Chem..

[25] Galasso M., Gambino S., Romanelli M.G., Donadelli M., Scupoli M.T. (2021). Browsing the oldest antioxidant enzyme: Catalase and its multiple regulation in cancer. Free Radic. Biol. Med..

[26] Glorieux C., Dejeans N., Sid B., Beck R., Calderon P.B., Verrax J. (2011). Catalase overexpression in mammary cancer cells leads to a less aggressive phenotype and an altered response to chemotherapy. Biochem. Pharmacol..

[27] Sander C.S., Hamm F., Elsner P., Thiele J.J. (2003). Oxidative stress in malignant melanoma and non-melanoma skin cancer. Br. J. Dermatol..

[28] Flor S., Oliva C.R., Ali M.Y., Coleman K.L., Greenlee J.D., Jones K.A., Monga V., Griguer C.E. (2021). Catalase Overexpression Drives an Aggressive Phenotype in Glioblastoma. Antioxidants.

[29] Heinzelmann S., Bauer G. (2010). Multiple protective functions of catalase against intercellular apoptosis-inducing ROS signaling of human tumor cells. Biol. Chem..

[30] Glorieux C., Buc Calderon P. (2024). Targeting catalase in cancer. Redox Biol..

[31] Putnam C.D., Arvai A.S., Bourne Y., Tainer J.A. (2000). Active and inhibited human catalase structures: Ligand and NADPH binding and catalytic mechanism. J. Mol. Biol..

[32] Feinstein R.N., Fry R.J., Staffeldt E.F. (1978). Carcinogenic and antitumor effects of aminotriazole on acatalasemic and normal catalase mice. J. Natl. Cancer Inst..

[33] Steinhoff D., Weber H., Mohr U., Boehme K. (1983). Evaluation of amitrole (aminotriazole) for potential carcinogenicity in orally dosed rats, mice, and golden hamsters. Toxicol. Appl. Pharmacol..

[34] Codenotti S., Faggi F., Ronca R., Chiodelli P., Grillo E., Guescini M., Megiorni F., Marampon F., Fanzani A. (2019). Caveolin-1 enhances metastasis formation in a human model of embryonal rhabdomyosarcoma through Erk signaling cooperation. Cancer Lett..

[35] Codenotti S., Marampon F., Triggiani L., Bonù M.L., Magrini S.M., Ceccaroli P., Guescini M., Gastaldello S., Tombolini V., Poliani P.L. (2021). Caveolin-1 promotes radioresistance in rhabdomyosarcoma through increased oxidative stress protection and DNA repair. Cancer Lett..

[36] Petragnano F., Pietrantoni I., Camero S., Codenotti S., Milazzo L., Vulcano F., Macioce G., Giordani I., Tini P., Cheleschi S. (2020). Clinically relevant radioresistant rhabdomyosarcoma cell lines: Functional, molecular and immune-related characterization. J. Biomed. Sci..

[37] Glorieux C., Zamocky M., Sandoval J.M., Verrax J., Calderon P.B. (2015). Regulation of catalase expression in healthy and cancerous cells. Free Radic. Biol. Med..

[38] Taniguchi M., Hashimoto M., Hori N., Sato K. (2005). CCAAT/enhancer binding protein-beta (C/EBP-beta), a pivotal regulator of the TATA-less promoter in the rat catalase gene. FEBS Lett..

[39] Luo D., Rando T.A. (2003). The regulation of catalase gene expression in mouse muscle cells is dependent on the CCAAT-binding factor NF-Y. Biochem. Biophys. Res. Commun..

[40] Liu Y., Adachi M., Zhao S., Hareyama M., Koong A.C., Luo D., Rando T.A., Imai K., Shinomura Y. (2009). Preventing oxidative stress: A new role for XBP1. Cell Death Differ..

[41] Yoshida H., Matsui T., Yamamoto A., Okada T., Mori K. (2001). XBP1 mRNA is induced by ATF6 and spliced by IRE1 in response to ER stress to produce a highly active transcription factor. Cell.

[42] Riha R., Gupta-Saraf P., Bhanja P., Badkul S., Saha S. (2017). Stressed Out-Therapeutic Implications of ER Stress Related Cancer Research. Oncomedicine.

[43] León-Annicchiarico C.L., Ramírez-Peinado S., Domínguez-Villanueva D., Gonsberg A., Lampidis T.J., Muñoz-Pinedo C. (2015). ATF4 mediates necrosis induced by glucose deprivation and apoptosis induced by 2-deoxyglucose in the same cells. FEBS J..

[44] Sabnis A.J., Guerriero C.J., Olivas V., Sayana A., Shue J., Flanagan J., Asthana S., Paton A.W., Paton J.C., Gestwicki J.E. (2016). Combined chemical-genetic approach identifies cytosolic HSP70 dependence in rhabdomyosarcoma. Proc. Natl. Acad. Sci. USA.

[45] Aghaei M., Nasimian A., Rahmati M., Kawalec P., Machaj F., Rosik J., Bhushan B., Bathaie S.Z., Azarpira N., Los M.J. (2021). The Role of BiP and the IRE1α-XBP1 Axis in Rhabdomyosarcoma Pathology. Cancers.

[46] McCarthy N., Dolgikh N., Logue S., Patterson J.B., Zeng Q., Gorman A.M., Samali A., Fulda S. (2020). The IRE1 and PERK arms of the unfolded protein response promote survival of rhabdomyosarcoma cells. Cancer Lett..

[47] Glorieux C., Auquier J., Dejeans N., Sid B., Demoulin J.B., Bertrand L., Verrax J., Calderon P.B. (2014). Catalase expression in MCF-7 breast cancer cells is mainly controlled by PI3K/Akt/mTor signaling pathway. Biochem. Pharmacol..

[48] Glorieux C., Sandoval J.M., Fattaccioli A., Dejeans N., Garbe J.C., Dieu M., Verrax J., Renard P., Huang P., Calderon P.B. (2016). Chromatin remodeling regulates catalase expression during cancer cells adaptation to chronic oxidative stress. Free Radic. Biol. Med..

[49] Konki M., Pasumarthy K., Malonzo M., Sainio A., Valensisi C., Söderström M., Emani M.R., Stubb A., Närvä E., Ghimire B. (2016). Epigenetic Silencing of the Key Antioxidant Enzyme Catalase in Karyotypically Abnormal Human Pluripotent Stem Cells. Sci. Rep..

[50] Margoliash E., Novogrodsky A., Schejter A. (1960). Irreversible reaction of 3-amino-1:2:4-triazole and related inhibitors with the protein of catalase. Biochem. J..

[51] Grytsai O., Valiashko O., Penco-Campillo M., Dufies M., Hagege A., Demange L., Martial S., Pagès G., Ronco C., Benhida R. (2020). Synthesis and biological evaluation of 3-amino-1,2,4-triazole derivatives as potential anticancer compounds. Bioorg. Chem..

[52] Tasia W., Washio A., Yamate K., Morita K., Mori Y., Kahar P., Sasaki R., Ogino C. (2025). Catalase-Knockout Complements the Radio-Sensitization Effect of Titanium Peroxide Nanoparticles on Pancreatic Cancer Cells. Molecules.

[53] Codenotti S., Marampon F., Megiorni F., Cattaneo C.G., Gastaldello S., Pozzo E., Sampaolesi M., Rota R., Keller C., Fanzani A. (2025). Disrupting resistance: Novel therapeutic approaches to combat multidrug resistance in fusion-negative rhabdomyosarcoma. Cancer Drug Resist..

[54] Versari I., Salucci S., Bavelloni A., Battistelli M., Traversari M., Wang A., Sampaolesi M., Faenza I. (2025). The Emerging Role and Clinical Significance of PI3K-Akt-mTOR in Rhabdomyosarcoma. Biomolecules.

[55] Prada E., Táboas P., Andrades E., Gómez-González S., Mateo-Lozano S., Cebria-Xart A., Berenguer-Molins P., Perera-Bel J., Arcon J.P., Saen-Oon S. (2025). PRKG1 hinders myogenic differentiation and predicts response to AKT inhibitor ipatasertib in Rhabdomyosarcoma. Nat. Commun..

[56] Fanzani A., Poli M. (2017). Iron, Oxidative Damage and Ferroptosis in Rhabdomyosarcoma. Int. J. Mol. Sci..

[57] Kasiappan R., Jutooru I., Mohankumar K., Karki K., Lacey A., Safe S. (2019). Reactive Oxygen Species (ROS)-Inducing Triterpenoid Inhibits Rhabdomyosarcoma Cell and Tumor Growth through Targeting Sp Transcription Factors. Mol. Cancer Res..

[58] Codenotti S., Poli M., Asperti M., Zizioli D., Marampon F., Fanzani A. (2018). Cell growth potential drives ferroptosis susceptibility in rhabdomyosarcoma and myoblast cell lines. J. Cancer Res. Clin. Oncol..

[59] Dächert J., Ehrenfeld V., Habermann K., Dolgikh N., Fulda S. (2020). Targeting ferroptosis in rhabdomyosarcoma cells. Int. J. Cancer.

[60] Selim O., Song C., Kumar A., Phelan R., Singh A., Federman N. (2023). A review of the therapeutic potential of histone deacetylase inhibitors in rhabdomyosarcoma. Front. Oncol..

[61] Cassandri M., Pomella S., Rossetti A., Petragnano F., Milazzo L., Vulcano F., Camero S., Codenotti S., Cicchetti F., Maggio R. (2021). MS-275 (Entinostat) Promotes Radio-Sensitivity in PAX3-FOXO1 Rhabdomyosarcoma Cells. Int. J. Mol. Sci..

[62] Marampon F., Di Nisio V., Pietrantoni I., Petragnano F., Fasciani I., Scicchitano B.M., Ciccarelli C., Gravina G.L., Festuccia C., Del Fattore A. (2019). Pro-differentiating and radiosensitizing effects of inhibiting HDACs by PXD-101 (Belinostat) in in vitro and in vivo models of human rhabdomyosarcoma cell lines. Cancer Lett..

[63] Enßle J.C., Boedicker C., Wanior M., Vogler M., Knapp S., Fulda S. (2018). Co-targeting of BET proteins and HDACs as a novel approach to trigger apoptosis in rhabdomyosarcoma cells. Cancer Lett..

[64] Bharathy N., Berlow N.E., Wang E., Abraham J., Settelmeyer T.P., Hooper J.E., Svalina M.N., Bajwa Z., Goros M.W., Hernandez B.S. (2019). Preclinical rationale for entinostat in embryonal rhabdomyosarcoma. Skelet. Muscle.

[65] O’Malley Y.Q., Reszka K.J., Rasmussen G.T., Abdalla M.Y., Denning G.M., Britigan B.E. (2003). The *Pseudomonas* secretory product pyocyanin inhibits catalase activity in human lung epithelial cells. Am. J. Physiol. Lung Cell. Mol. Physiol..

[66] Krych J., Gebicka L. (2013). Catalase is inhibited by flavonoids. Int. J. Biol. Macromol..

[67] Durner J., Klessig D.F. (1996). Salicylic acid is a modulator of tobacco and mammalian catalases. J. Biol. Chem..

[68] Dong P.T., Jusuf S., Hui J., Zhan Y., Zhu Y., Liu G.Y., Cheng J.X. (2022). Photoinactivation of catalase sensitizes a wide range of bacteria to ROS-producing agents and immune cells. JCI Insight.

